# A [^11^C]PBR28 PET study on the associations between sleep health and microglial density

**DOI:** 10.1186/s12974-025-03613-1

**Published:** 2025-11-14

**Authors:** Leonie JT Balter, Jonatan Malmros, Per Stenkrona, Andrea Varrone, Anton Forsberg, Erik Gustavsson, Cedrique E. Mouyobo, Grégoria Kalpouzos, Goran Papenberg

**Affiliations:** 1 Department of Clinical Neuroscience, Karolinska Institutet, Stockholm, 171 65 Sweden; 2 Department of Psychology, Stress Research Institute, Stockholm University, Stockholm, 114 19 Sweden; 3 Department of Psychiatry, Radboud University Medical Centre, Nijmegen, 6525 GA The Netherlands; 4 Donders Institute for Brain, Cognition and Behaviour, Radboud University, Nijmegen, 6525 EN The Netherlands; 5 Department of Medical Sciences, Clinical Psychiatry, Uppsala University, Uppsala, 751 85 Sweden; 6 Department of Clinical Neuroscience, Centre for Psychiatry Research, Region Stockholm, Karolinska Institutet, & Stockholm Health Care Services, Stockholm, 171 77 Sweden; 7 Aging Research Center, Department of Neurobiology, Care Sciences and Society, Karolinska Institutet and Stockholm University, Stockholm, 171 77 Sweden

**Keywords:** Sleep health, Sleep duration, Neuroinflammation, Microglial density, TSPO, PET, Inflammation

## Abstract

Sleep disturbances and inflammation are interconnected through shared regulatory mechanisms and are both implicated in age-related diseases. However, their connection at the level of brain-specific inflammation remains underexamined in humans. This study investigated whether specific dimensions of sleep are associated with microglial density, as measured by translocator protein (TSPO) levels, a biomarker of neuroinflammation. TSPO levels were measured using a single [^11^C]PBR28 positron emission tomography (PET) scan in 39 healthy adults aged 50–81 years (M_age_ = 66.7, SD = 8.9; 19 females, 20 males). Sleep dimensions were assessed using the Karolinska Sleep Questionnaire on three occasions over five years, twice before and once around the time of PET imaging. Shorter sleep, more frequent napping, daytime fatigue, and sleep insufficiency were associated with higher TSPO levels in the middle frontal cortex (MFC). Conversely, longer sleep was associated with higher TSPO levels in the hippocampus and putamen. Exploratory factor analysis and bootstrapping confirmed a negative association between a factor representing shorter sleep and MFC TSPO levels. Additionally, a greater deviation from optimal sleep duration over the five years, in either direction from eight hours, was associated with higher current TSPO levels in all but one examined brain regions. Peripheral C-reactive protein levels did not significantly correlate with the sleep variables or TSPO levels in any of the brain regions. Analyses were adjusted for age and sex. These findings suggest that insufficient and prolonged sleep durations are associated with elevated microglial density in frontostriatal and limbic systems, respectively, among healthy middle-aged and older adults, without any associations with peripheral inflammation. Further longitudinal studies are needed to clarify directionality and whether changes in sleep duration over time may serve as early indicators of brain health.

**Supplementary Information:**

The online version contains supplementary material available at 10.1186/s12974-025-03613-1.

## Introduction

Inflammation and sleep share a close relationship through shared regulatory mechanisms. A large body of evidence highlights their bidirectional relationship and co-occurrence [1]. For example, sleep disturbances are common features of chronic inflammatory conditions, including autoimmune and metabolic disorders [2], and peripheral low-grade inflammation is common in sleep disorders [3]. Immune signaling plays a fundamental role in the regulation of sleep. Several inflammatory cytokines, most notably interleukin-1 beta (IL-1β) and tumor necrosis factor alpha (TNF-α), are often referred to as “sleep-regulatory substances” [4]. Their expression increases in response to extended wakefulness. Human and animal work show that elevations in cytokines, when triggered by infection, exogenous immune stimulation, or sleep deprivation, generally promote increases in total sleep duration, longer non-rapid eye movement (non-REM) sleep duration and increased slow-wave activity, often with concurrent reductions in REM sleep and reduced sleep continuity [1, 4–8]. These effects may reflect an adaptive mechanism to promote recovery through an increased sleep drive, but also signal underlying pathophysiology.

Animal studies show that sleep deprivation enhances the expression of IL-1β and TNF-α mRNA in brain regions involved in sleep regulation, including the frontal cortex, hypothalamus, hippocampus, basal forebrain, and pons in rodents [4, 9–11]. Long-term sleep fragmentation has also been linked to microglia-mediated neuroinflammation in mice, predominantly in the hippocampus [12]. Given this evidence, investigating region-specific patterns of brain immune activity, such as in brain areas involved in sleep-wake regulation and those sensitive to aging, may provide important insights beyond global assessments.

Positron Emission Tomography (PET) imaging using radioligands targeting 18 kDa translocator protein (TSPO) is a widely used tool to quantify central nervous system (CNS) glial cells in vivo and is considered an index of microglial density [13–15]. The expression of TSPO is upregulated in reactive microglia cells – the CNS’s resident immune cells that play a key role in initiating and propagating neuroinflammatory processes [16]. TSPO availability in healthy individuals correlates with biological factors that are associated with low-level peripheral inflammation and altered sleep, such as older age (positive correlation) and high body mass index (both a negative and positive correlation) [17, 18]. Nevertheless, studies examining associations between sleep parameters and TSPO availability remain limited. In a study of 18 individuals with severe allergic disease, none of the assessed sleep dimensions correlated with TSPO binding despite patients having higher peripheral levels of TNF-α and IL-5 (but not IL-6, IL-8, or interferon-gamma (IFN-γ)), and worse sleep during pollen season compared to other times of the year [19]. Expanding the sample to include 18 patients with rheumatoid arthritis also revealed no discernible correlations between TSPO binding and objective or subjective sleep [20]. These studies assessed global TSPO binding and did not formally assess TSPO levels in brain areas involved in sleep regulation. Given the scarcity of studies and generally small sample sizes, larger and more diverse cohorts are needed to further examine the relationships between individual differences in sleep dimensions and biomarkers of neuroinflammation.

The multifaceted nature of sleep [21] presents challenges in identifying the central aspects of sleep for inflammation and health. Sleep encompasses dimensions such as sleep duration, timing, quality, continuity, consistency, and social jetlag (i.e., difference in sleep timing between workdays and free days), all of which have implications for health outcomes [22–24]. However, sleep as a multidimensional construct is often not fully considered [25]. Sleep dimensions such as sleep apnea symptoms, later sleep timing, and social jetlag have been associated with elevated peripheral inflammation [26, 27]. Meta-analyses of observational and experimental studies show that chronic sleep disturbances, multiple nights of sleep restriction, and long sleep duration (>8 h) are associated with higher levels of peripheral inflammatory markers, though these effects vary by biomarker [28–30]. However, acute (single night) sleep restriction does not reliably increase markers like C-reactive protein (CRP), IL-6, or TNF-α [28, 30], suggesting that short sleep may need to occur over an extended period to induce systemic inflammation [31]. Longitudinal studies support this, showing that persistently short sleep is associated with higher levels of peripheral inflammation over time [32–34] and that changes in sleep duration (both shorter and longer) are linked to adverse health outcomes [34]. Together these data emphasize the importance of assessing both current and sleep patterns over time across multiple sleep dimensions within the same individual, to understand which sleep characteristics most closely relate to brain immune processes.

In the current study, we used [^11^C]PBR28 to quantify microglial density (TSPO levels) as a biomarker of neuroinflammation in 39 healthy middle-aged and older individuals (i.e., no history of neurological or psychiatric conditions). To elucidate the dimensions of sleep that have the most significant relationship with neuroinflammation, we examined individual differences in self-reported sleep dimensions, including sleep quality, non-restorative sleep, daytime fatigue, sleep insufficiency, sleep need, napping behavior, chronotype, sleep duration, and social jetlag. We also assessed whether changes in sleep over a ~ 5-year period predicted current TSPO levels. We hypothesized that more pronounced sleep disturbances, such as poor sleep quality, would be associated with elevated TSPO levels, and that a decline in sleep quality and sleep duration over a 5-year period would be associated with higher TSPO levels. Secondary aims were to assess the correlation between plasma CRP levels and sleep dimensions, as well as the correspondence between TSPO binding and plasma CRP levels. No a priori hypotheses were specified regarding regional TSPO binding in relation to sleep.

## Methods and materials

### Participants

Thirty-nine participants (19 females, 20 males; mean age = 66.7 years; SD = 8.9; range 50–81) completed self-reported sleep assessments at three timepoints across ~ 5-year period (T0: Jan 2017-Dec 2017, T1: Nov 2019-Nov 2020 T2: Nov 2021-Oct 2022). The mean time difference in days between T0 and T1 was 983 days (range: 865 to 1100), and 733 days (range: 451 to 972) between T1 and T2. The mean time difference between T0 and T2 was 1715 days (range: 1415 to 1997; ca. 4.7 years). See Table 1 for an overview of descriptive information. PET TSPO imaging was performed at one timepoint only (T2). At T2, 24 participants were retired and 15 were working. At T0, 19 participants were retired, and at T1, 22 participants were retired.

**Table 1:** Descriptive information of sample demographics

Demographic variable	
Sex, *n*	
Male	20
Female	19
Age, *M* (*SD*)	66.7 years (8.9)
Range	50–81
BMI, *M* (*SD*)	24.9 (2.4)
Range	19.2–29.4
Body fat %, *M* (*SD*), range	
Male	22.3% (5.1), 12.5–29.6
Female	35.9% (4.4), 26.0–42.2
Muscle mass %, *M* (*SD*), range	
Male	33.6% (2.7), 30.0–38.8
Female	26.6% (1.9), 22.4–30.0
TSPO polymorphism (rs6971), *n*	
Mixed affinity binding	20
High affinity binding	19

*BMI* Body Mass Index

Key inclusion criteria were as follows: previous participation in the IronAge Study (T0 and T1), which focused on assessing brain iron content and accumulation in a large participant pool [35] (but also sleep and metabolomics [36]) and consent to be contacted for follow-up studies; 50 years or older; not carrying the genotype of a TSPO polymorphism (rs6971) associated with low binding to the TSPO radioligand [37]. In the current study, 19 (8 females, 11 males) had a high affinity (GG) binding phenotype and 20 (11 females, 9 males) had a mixed affinity (AG) binding phenotype. The total distribution volume (V_T_) for gray matter (GM) was *M =* 2.5 (*SD* = 0.5) in the AG group and *M* = 5.3 (*SD* = 1.2) in the GG group. These findings align with previous studies, such as one reporting V_T_ values of 2.6 and 4.3, respectively, in a younger cohort with a mean age of 23.9 [38]. Additional inclusion criteria were fluency in Swedish, right-handedness, and no history of neurological or psychiatric conditions. The study was approved by the Regional Ethics Committee and the Swedish Radiation Safety Authority. Participants provided written informed consent.

### General procedures

PET data were collected as part of a broader study investigating the potential link between brain iron and neuroinflammation. For the current study, sleep was assessed using a repeated measures design. The Karolinska Sleep Questionnaire (KSQ) was completed at T0, T1, and T2. Blood was collected for genetic analysis and for markers of iron and inflammation levels. TSPO PET imaging was performed at T2 only. Participants first underwent MRI assessment, followed by PET imaging and then blood sampling. Given the large number of questionnaires administered, the KSQ was distributed during the MRI visit and collected at the time of blood sampling. This allowed participants to complete all questionnaires at home during the study. The median interval between PET and blood collection was 36 days (range 4–126 days).

### Self-reported sleep measures

As per pre registration (10.17605/OSF.IO/DM2UB), information on various sleep dimensions was obtained using the Karolinska Sleep Questionnaire (KSQ). The KSQ is a validated and widely used instrument for obtaining sleep-related information [39]. The KSQ contains four items that refer to sleep quality (items referring to insomnia symptoms such as difficulties falling asleep and repeated awakenings), three items focusing on sleep apnea (involving aspects of snoring and cessation of breathing), three items capturing non-restorative sleep experiences (items referring to not feeling rested on awakening), and five items addressing daytime fatigue (items referring to sleepiness and fatigue during the daytime). Response options range from 0 “never” to 5 “always”, unless otherwise indicated. Item scores for these sleep dimensions were averaged. Higher scores indicate worse sleep. Other assessed sleep dimensions include: sleep insufficiency, assessed using the item “do you consider that you get enough sleep”, rated on 5-point scale from 0 “yes, definitely enough” to 4 “No, definitely not enough”; sleep need (in hours); napping frequency (5-point scale from 0 “almost never” to 4 “almost every day”); chronotype (5-point scale from 0 “extreme morning-type” to 4 “extreme evening-type”); average weekly sleep duration (average of sleep duration during workdays and free days, calculated using bedtime, wake-up time, and minutes to fall asleep); and social jetlag (difference between midpoint of sleep on free days and workdays). As per preregistration, the items “nap duration” and “nightmares” were not included because of conceptual overlap with other items and/or expected limited variability. Asleep quality item was not included as it was represented through a composite of four sleep disturbance items as described above.

### Quantification of TSPO binding

Participants were scanned at the Stockholm University Brain Imaging Centre using a Siemens 3-Tesla Prisma scanner equipped with a 20-channel head coil. PET data were acquired with a high-resolution research tomograph (HRRT; Siemens/CTI) over 60 min for [^11^C]PBR28 examination. T1-weighted structural MRI images were acquired for region-of-interest (ROI) definition, and kinetic modeling of PET data was performed to estimate total distribution volume (V_T_) of TSPO binding [38]. ROIs included global gray matter, caudate, frontal cortex, middle frontal cortex (MFC), thalamus, hippocampus, anterior cingulate cortex (ACC), insula, putamen, and brain stem. ROI selection was based on three main criteria: (1) relevance to sleep/wake regulation; (2) consistent TSPO binding in prior PET literature; and (3) relevance to inflammation-related pathophysiology. See Supplement for detailed information about imaging and analysis protocols.

### Genotyping

Blood samples at T0 were used to analyze the single nucleotide polymorphism in the TSPO gene (rs6971), which is known to affect [^11^C]PBR28 binding [37, 40]. See Supplement for detailed methods.

### Peripheral CRP levels

To quantify high-sensitivity CRP levels, plasma blood samples were analyzed using standard procedures. For the current analysis, only CRP measurements from T2 were included. Venous blood was collected before 10 AM while fasting since 8 PM the day before. Plasma samples were brought to the Centre for Clinical Laboratory Studies for immediate analyses (Karolinska Hospital, Stockholm).

### Statistical analyses

#### Relationships between TSPO binding, CRP, and self-reported sleep dimensions

Relationships between TSPO binding, peripheral CRP, and the self-reported sleep dimensions were assessed through linear fixed effect regression models. To allow for comparison of coefficients, the sleep measures were z-transformed before analyses.

#### Self-reported sleep changes across time (T0-T2) and TSPO binding (T2)

Linear mixed effect models were fitted to assess the relationships between self-reported sleep changes over time and TSPO binding at T2 using the *lmer* function of the lme4 R package [41]. All reported p-values are uncorrected. Given that TSPO PET was only acquired at T2 and sleep data were collected longitudinally at T0, T1, and T2, sleep served as the dependent variable, with TSPO ROI values treated as time-invariant predictors. A random intercept for participant ID was included. Time x ROI interactions were of primary interest to examine whether regional TSPO binding at T2 was associated with differing trajectories of sleep over time. All models that involved regional TSPO binding included global TSPO levels in gray matter (i.e., volume of distribution; V_T_) as a variable in the model. The model assessing global gray matter corrected for TSPO genotype. All models included sex (male serving as reference) and age as fixed effect predictors. For PET-related analyses, we additionally adjusted for the interval (days) between the PET scan and blood sampling during which sleep data were collected, to account for temporal discrepancies. However, it should be noted that participants could have completed the questionnaire data at any time between the PET scan and blood sampling. Longitudinal analyses further included retirement status as a time-varying covariate.

#### Data exclusion and missing data

For CRP values, one datapoint of 7.5 mg/L was excluded due to deviating more than four standard deviations from the sample’s mean. One participant worked night shifts at T1 and T2; their T1 and T2 data on sleep duration, sleep duration deviation, and social jetlag data at these timepoints were therefore excluded. The sleep apnea variable was excluded due to minimal variation (see Supplement Figure S1c).

#### Deviations from preregistration and additional analyses

##### Bootstrapping

As per preregistration, we did not apply multiple comparisons correction. To derive more robust estimates of confidence intervals, we supplemented the analyses assessing the relationships between TSPO binding, CRP, and sleep dimensions with a bootstrap sample of 1,000 samples using re-sampling with replacement. The *Boot* function of the car R package was used for this purpose. This approach allows testing the stability of the results while minimizing the risk of overcorrection.

##### Latent sleep factors

To examine whether the sleep-TSPO associations were robust to shared variance among interrelated sleep measures, we conducted a post-hoc exploratory factor analysis (EFA) on the sleep variables using the *fa* function of the *psych* R Package. This step deviates from the pre-registration and functions as a robustness check. This finding should not be interpreted as a new result but as convergent evidence, corroborating the primary item-level analyses. For further details, see Supplementary Material.

##### Sleep duration deviation

The relationship between health and sleep duration often displays a U-shaped relationship, meaning that both shorter and longer sleep duration are associated with worse health [42, 43]. To accommodate potential U-shaped relationships, we computed sleep duration deviation, defined as the absolute deviation from the recommended eight hours of sleep per night for healthy adults [44]. Higher values reflect either shorter or longer sleep duration than the recommended eight hours. The sample’s mean sleep duration at each timepoint was 8h12, 8h04 and 8h17min, respectively.

##### Baseline model and covariates

In the pre-registration we outlined using a forward stepwise model comparison approach including sex and age, with model complexity increasing with one variable at a time. However, we decided to include sex and age in all models to reduce the number of analyses. In longitudinal analyses we further adjusted for changes in retirement status and in PET-related analyses for the interval (days) between the PET scan and blood sampling, during which the sleep data were collected.

##### Regions of interest

Reliability estimation for smaller ROIs has not been performed and smaller ROIs, such as the anterior insula and hypothalamus, were not included in any of the analyses.

## Results

### Correlations between TSPO levels and self-reported sleep dimensions

**Fig. 1:**
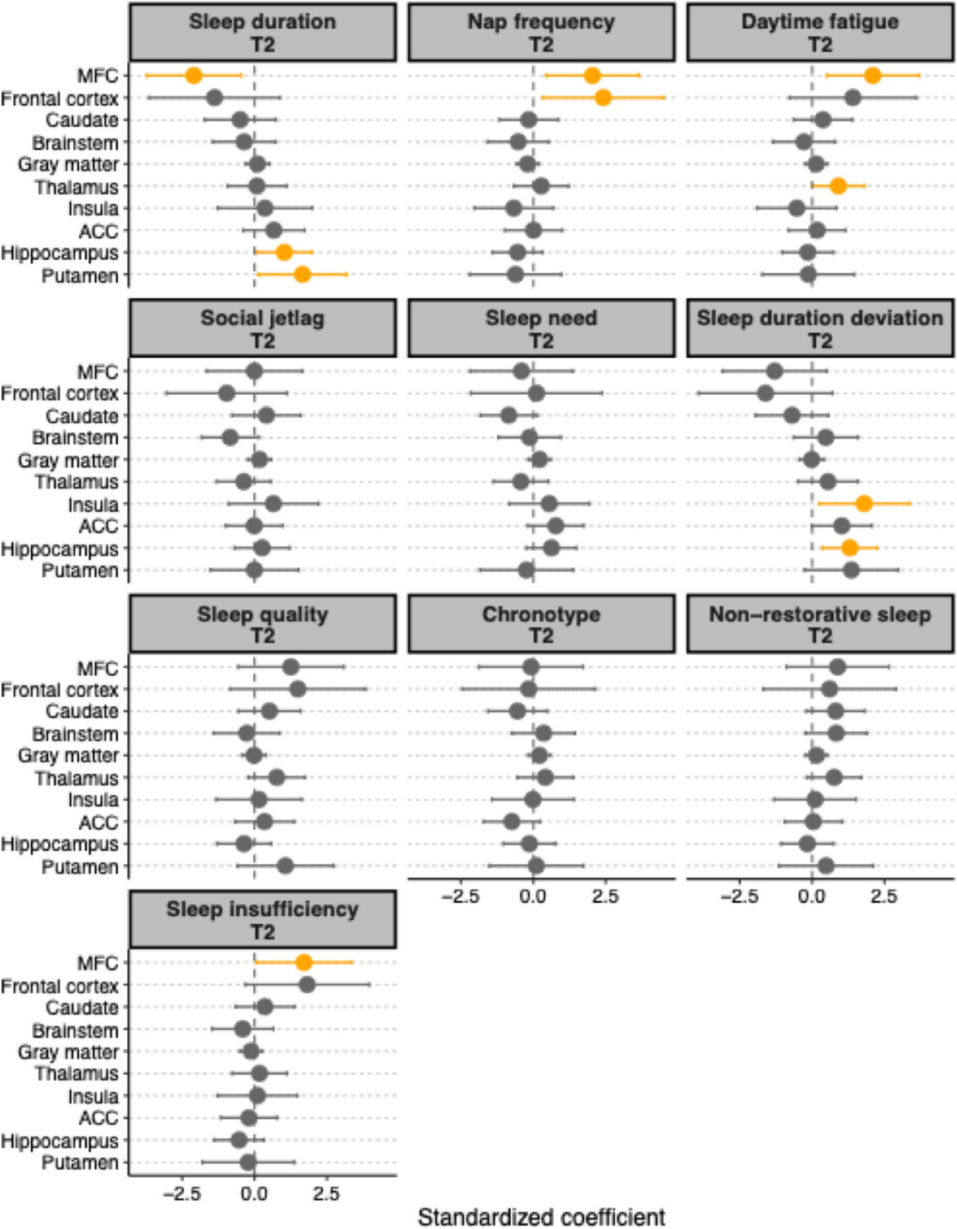
Coefficient plots illustrating the relationships between standardized sleep features and TSPO levels in regions of interest (ROI) (y-axes). Orange circles indicate statistically significant results (*p* <.05) and gray circles indicate statistically non-significant results (*p* >.05). Error bars represent 95% confidence intervals. A coefficient of 0 indicates no relationship between the sleep dimension and TSPO level in the respective ROI. Higher chronotype values indicate greater eveningness; Higher values of sleep duration deviation indicate either a shorter or longer sleep duration eight hours. See Supplement Figure S5 for residual plots of the significant associations. MFC = Middle Frontal Cortex; ACC = Anterior Cingulate Cortex

As shown in Fig. 1, shorter sleep duration was associated with higher TSPO binding level in the MFC (b = −2.09, 95% CI [−3.72, −0.46], *p* =.013; bootstrapped 95% CI [−3.82, −0.35]). In contrast, higher TSPO binding level in the putamen (b = 1.66, 95% CI [0.13, 3.19], *p* =.035; bootstrapped 95% CI [0.25, 3.16]) and hippocampus (b = 1.03, 95% CI [0.08, 1.98], *p* =.035, bootstrapped 95% CI [0.01, 2.46]) were associated with longer sleep duration. Higher TSPO level in the MFC was also associated with more frequent naps (b = 2.04, 95% CI [0.44, 3.65], *p* =.014; bootstrapped 95% CI [0.93, 3.87]), more daytime fatigue (b = 2.10, 95% CI [0.50, 3.69], *p* =.011; bootstrapped 95% CI [0.65, 3.53]) and sleep insufficiency (b = 1.71, 95% CI [0.06, 3.35], *p* =.042; bootstrapped 95% CI [0.54, 3.19]). Additionally, frontal cortex TSPO level was also associated with more frequent naps (b = 2.42, 95% CI [0.34, 4.50], *p* =.024; bootstrapped 95% CI [0.54, 5.43]) and thalamus TSPO level with daytime fatigue (b = 0.91, 95% CI [0.02, 1.80], *p* =.046; but not bootstrapped 95% CI [−0.07, 1.93]). A greater deviation in sleep duration (either shorter or longer than eight hours of sleep) was associated with higher TSPO binding level in the hippocampus (b = 1.30, 95% CI [0.37, 2.24], *p* =.008; bootstrapped 95% CI [0.14, 2.46]) and insula (b = 1.80, 95% CI [0.24, 3.35], *p* =.025; but not bootstrapped 95% CI [−0.56, 3.05]). Detailed results can be found in Supplementary Materials Table S3. Distribution plots of the sleep dimension variables and TSPO binding ROIs are presented in the Supplementary Materials Figure S1 and S2.

These findings suggest that dimensions more closely related to the duration of sleep (sleep duration, napping frequency, sleep duration deviation, sleep insufficiency) may have a stronger association with TSPO levels than other aspects of sleep.

### Sleep factors and TSPO levels

Although the scree plot and parallel analysis suggested a one-factor solution. theoretical frameworks and empirical findings (e.g [21, 45]) indicate that sleep duration is partially dissociable from other sleep dimensions. Given the goal of examining result stability, we extracted two factors.

**Fig. 2:**
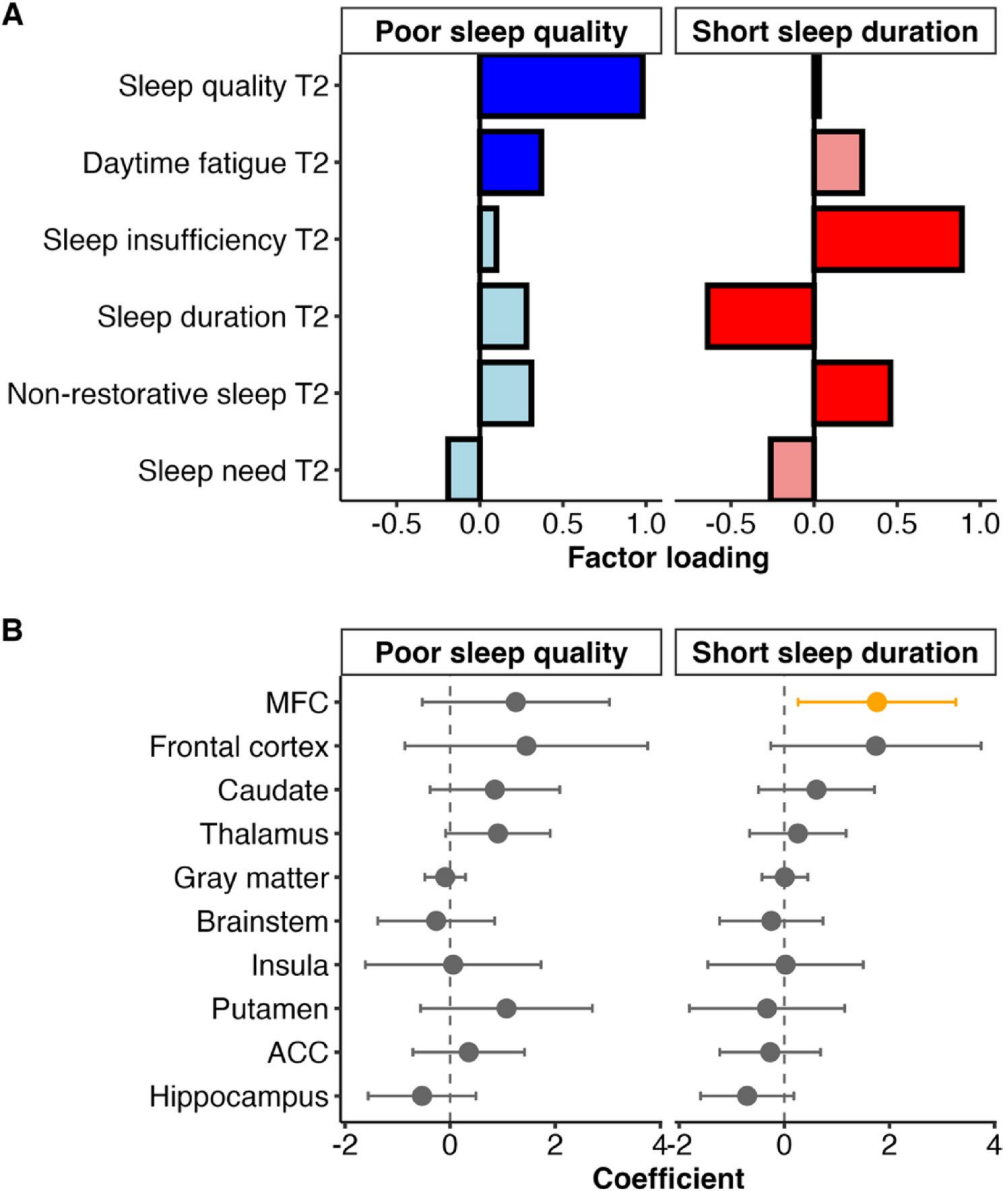
Factor analysis of the sleep dimension variables and relationships with sleep TSPO levels. **A** Each panel shows the factor loadings of the sleep factors. Higher factor loadings correspond to stronger loadings for the respective factor, with both positive and negative loadings. More saturated colored bars indicate the sleep items that load ≥ 0.30 and load strongest on the respective factor. Labelling of the factors is based on the sleep items that load most strongly on each respective construct. **B** Unstandardized coefficient plot of the relationship between TSPO binding level in each ROI and each factor as obtained from (**A**). 0 = no relationship. A positive coefficient indicates that TSPO binding level is associated with shorter sleep duration. Error bars represent 95% confidence intervals. Yellow dots indicate statistically significant relationships (*p* <.05), gray dots indicate statistically non-significant relationships (*p* >.05). MFC = Middle Frontal Cortex; ACC = Anterior Cingulate Cortex

As shown in Fig. 2a, the first factor reflects poor sleep quality and the second factor captures short sleep duration. Each factor is the weighted combination of all sleep variables. It includes shared variance across multiple sleep-related variables, including those with weaker contributions. The correlation between the two extracted factors was *r* =.55. Consistent with the analysis of the individual sleep variables, higher TSPO binding level in the MFC (b = 1.76, 95% CI [0.20, 3.32], *p* =.028; bootstrapped 95% CI [0.62, 3.15]) was associated with the shorter sleep duration factor. None of the ROIs was associated with the poor sleep quality factor (see Fig. 2b).

### Relationships of CRP with TSPO levels and CRP with sleep dimensions

**Fig. 3:**
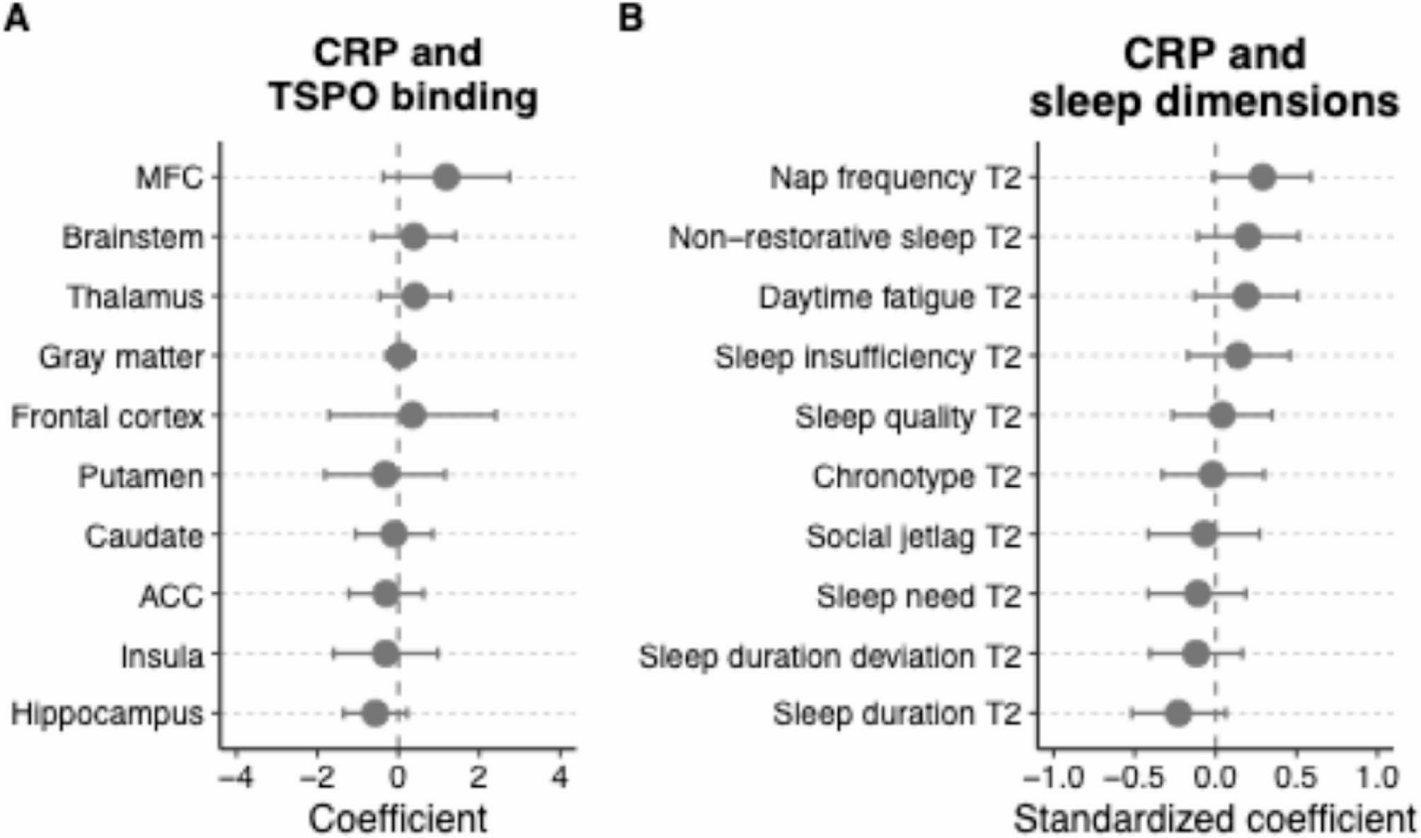
Coefficient plots showing the relationships of CRP with TSPO binding and sleep dimensions. **A** CRP level and TSPO binding in regions of interest (ROIs). The coefficients are unstandardized. **B** CRP level and sleep dimensions. Sleep dimension variables are standardized to allow for comparison of coefficients. Higher chronotype values indicate being a stronger evening chronotype. Higher values of sleep duration deviation indicate either a shorter or longer sleep duration than eight hours. MFC = Middle Frontal Cortex; ACC = Anterior Cingulate Cortex

The average plasma CRP level in this sample was 1.23 mg/L (*SD* = 0.89, range [0.3–3.9]). Plasma CRP level did not significantly correlate with age (b = 0.00, 95% CI [−0.03, 0.04], *p* =.798) and there were no sex differences (b = −0.29, 95% CI [−0.88, 0.29], *p* =.313). As show in Fig. 3a, plasma CRP level did not correlate with TSPO level in any of the ROIs. Plasma CRP level was not significantly correlated with sleep duration (b = −0.23, 95% CI [−0.52, 0.06], *p* =.121 although bootstrapped 95% CI was − 0.48, −0.02) (see Fig. 3b and Supplement Table S4 and S5).

### Sleep changes across timepoints and relationships with TSPO levels

At the group level, the sleep dimension variables remained stable across the ~ 5-year period (from T0 to T2), with the exception of an improvement in non-restorative sleep (i.e., feeling more refreshed upon waking) at T2 compared to T0 (b = −0.26, 95% CI [−0.52, 0.00], *p* =.050, adjusted for retirement status). A larger change in sleep duration deviation across timepoints was associated with higher TSPO levels at T2 in all ROIs except for the frontal cortex (95% CI [0.00, 0.23]), indicating that individuals whose sleep duration deviated more from optimal sleep over the 5-year period (i.e., sleep duration that became short or longer than the recommended eight hours) had higher TSPO levels at T2 (adjusted for sex, age, retirement status, and TSPO gene (for gray matter) or gray matter volume of distribution (VT) (for all other ROIs). None of the other sleep dimensions were associated with TSPO levels at T2. Detailed results can be found in Supplementary Materials Figure S6-7 and Table S7.

## Discussion

The current study investigated associations between multiple dimensions of sleep health and brain TSPO levels in adults aged 50–81. Sleep duration showed the most consistent association with TSPO levels across different brain regions. Specifically, shorter sleep was associated with higher TSPO levels in the MFC, whereas longer sleep duration was associated with higher TSPO levels in the hippocampus and putamen. Additionally, higher TSPO level in the MFC correlated with more frequent napping, greater daytime fatigue, and increased sleep insufficiency. Follow-up analyses using exploratory factor analysis reinforced the predominant association between shorter sleep/insufficiency and TSPO levels in the MFC, but not between longer sleep and TSPO levels in the hippocampus and putamen. Contrary to hypothesis, sleep quality did not show significant associations with TSPO binding in any of the examined brain regions. Longitudinal sleep analysis further showed that a greater change in sleep duration deviation over five years (whether toward shorter or longer durations than the eight-hour benchmark) was associated with higher TSPO levels in most brain regions at follow-up (except for the frontal cortex). This suggests that sustained deviations from optimal sleep duration, in either direction, may cumulatively contribute to neuroinflammatory processes over time. That these longitudinal associations were more widespread than the cross-sectional analysis at T2 could relate to that a single timepoint sleep duration captures more momentary, state-like variation. In addition, sleep duration and sleep duration deviation represent somewhat different constructs. Sleep duration reflects the absolute amount of sleep, whereas the deviation metric quantifies the degrees of deviation from optimal sleep, collapsing shorter and longer sleep into a single index of non-optimality. The latter effectively models the U-shaped relationship of sleep duration with health outcomes. These metrics may also engage partially different physiological pathways. Short sleep may, for example, index heightened stress reactivity [46], whereas long sleep may reflect increased physiological sleep need or pressure due to, for example, underlying morbidity [47], processes that the deviation metric pools into a single metric. Indeed, that any deviation from the recommended eight hours sleep duration over time was associated with a biomarker of brain inflammation aligns with prior reports of U-shaped sleep duration-health relationships, including elevated risk of cardiovascular diseases, metabolic disorders, and mortality [43], as well as markers of amyloid deposition in cerebrospinal fluid (CSF) [48]. Large-scale multi-modal imaging data have similarly reported that deviations from recommended sleep durations are associated with indicators of poorer brain health such as white matter integrity and hippocampal volume [49, 50]. Our findings extend this literature by showing that individual variation in neuroinflammation, a hallmark mechanism of aging, may contribute to some of these associations [51].

No significant associations between plasma CRP and TSPO PET signal were observed, supporting the notion that peripheral levels of CRP cannot be assumed to reflect central neuroimmune processes. This aligns with several TSPO PET studies using [¹¹C]PBR28, which have reported weak, absent, or inverse correlations between peripheral inflammatory markers and central TSPO binding [52–56]. This highlights the importance of using direct CNS measures such as TSPO PET to assess brain-specific inflammation.

TSPO levels in the MFC exhibited the most consistent relationships with sleep dimensions (i.e., sleep duration, daytime fatigue, napping frequency, and sleep insufficiency). Sleep duration and daytime fatigue are often intertwined, as short sleep duration can contribute to daytime fatigue and sleepiness (for a review see [57]). In the current sample the correlation between sleep duration and daytime fatigue was *r*_s_ = −0.31, *p* =.069. TSPO levels in the MFC also showed positive associations with napping frequency and sleep insufficiency, a convergent pattern consistent with the idea that insufficient sleep increases need for recovery, where greater napping behavior may be (i) a compensatory behavior for insufficient nocturnal sleep [58] (consistent with the MFC associations for shorter sleep, fatigue, and sleep insufficiency), and/or (ii) greater sleep need related to low-grade neuroinflammatory processes. The MFC is particularly susceptible to fatigue, with studies showing compromised functionality under fatigued conditions [59]. While the exact mechanisms remain speculative, one possibility is that shorter sleep increases neuroinflammation in the MFC, thereby disrupting its normal functioning and contributing to the experience of fatigue. MFC’s role in regulating a number of cognitive processes, including cognitive control and emotional regulation [60], may also make it more vulnerable to the effects of fatigue. The distinct associations observed between TSPO levels and sleep duration in frontostriatal versus limbic systems suggest that TSPO levels in different neural systems may be differentially related to sleep duration. Specifically, higher TSPO levels in the MFC were associated with shorter sleep, while higher TSPO levels in limbic areas, including hippocampus and putamen, were associated with longer sleep duration. Longer sleep duration has been linked to an increased risk of dementia [61]. This aligns well with our findings in the hippocampus, a region critically involved in Alzheimer’s disease. Furthermore, there is well-documented evidence of putamen integrity decline in Parkinson’s disease (PD) [62], along with increased TSPO levels [63], and recent findings from the UK biobank linking long sleep duration to an increased risk of PD [64]. The observed association between TSPO in the putamen and long sleep duration reflects a similar pattern in healthy adults. Future studies could examine whether these associations between TSPO levels and sleep duration serve as indicators of neurodegenerative risk.

Although long sleep duration is often associated with adverse health, it is unlikely that long sleep duration itself causes poor health [65]. Instead, it may reflect presence of an underlying health condition, such as early-stage (undiagnosed) pathology, asymptomatic infections, or subclinical psychiatric issues [47, 66]. Although our sample was screened for various health conditions (e.g., neuropsychiatric and neurodegenerative diseases), it is possible that some individuals had underlying early-stage pathology that contribute to (neuro)inflammation and/or a longer sleep duration [61]. Similarly, differences in biological aging could play a role, influencing sleep patterns and inflammatory processes [50, 67].

Sleep serves critical neuroprotective functions for the brain. Beyond metabolic clearance [68] (but also see [69]) and synaptic downscaling to energetically sustainable levels [70, 71], sleep drives immune-supportive functions [1, 72]. Experimental studies in rodents have shown that acute and prolonged sleep deprivation lead to an increase in inflammatory markers in the brain [4, 9–11]. Human studies on sleep and neuroinflammation are limited. A pilot study with 18 patients with severe allergy [19], and a subsequent study adding 18 patients with rheumatoid arthritis [20] found no significant associations between TSPO levels and sleep metrics. Their studies focused on global TSPO levels in gray matter and examination of a different population. Notably, global TSPO levels were also unrelated to the sleep dimensions in our data, suggesting that region-specific TSPO levels may need to be considered. Future longitudinal research could investigate whether low level region-specific variations in neuroimmune activity among middle-aged and older individuals predict later cognitive decline or neurodegenerative risk.

Several limitations should be noted. First, the reliance on self-reported sleep data may have introduced bias. Future studies may incorporate measures such as actigraphy or polysomnography. Additionally, the sample consisted of healthy middle-aged and older adults, potentially limiting variability in certain sleep dimensions and (neuro)inflammation. This, together with the moderate sample size, may have reduced statistical power to detect relationships with TSPO levels, except for sleep duration which naturally varies more between people. Relatedly, and contrary to hypothesis, sleep quality was not significantly associated with TSPO in any region. An interaction between sleep duration and quality is also plausible, where adverse effects of sleep duration primarily occur primarily when accompanied by poor sleep quality [42]; however our study was not powered to test such effects. Other limitations include the uncertainty regarding the exact time of sleep assessment, as participants had the possibility to fill out questionnaires at any timepoint between MR assessment and blood sampling (median interval = 36 days, range: 4–126), and the limited cellular specificity of TSPO PET radioligands. In humans, the TSPO signal is thought to reflect microglial density more than microglial activation [15], meaning that an elevated TSPO signal does not directly equate to an active inflammatory process. That said, increases in microglial density are known to precede microglial activation in various disease states [73, 74]. Our approach of analyzing each sleep dimension separately enabled us to identify which specific aspects of sleep are associated with neuroinflammation, but this approach also increased the risk of Type I errors. Instead of applying multiple comparisons correction, we used bootstrapping and additional analyses using exploratory factor analysis to assess the robustness of our findings, where relationships between MFC and sleep duration are most dominant. While some observed effects may be attributable to chance, the consistent associations with sleep duration (including its relationship with TSPO levels in the MFC, and 5-year change in sleep duration deviation) support the robustness of our findings. This raises the possibility that stable, balanced sleep duration helps sustain neuroimmune homeostasis in aging. Such a relationship would position sleep duration as a modifiable correlate of brain immune health, offering a potential avenue for preventive intervention. Nevertheless, these results should be interpreted as hypothesis-generating and warrant further validation in future studies. Lastly, although PET imaging was conducted at a single timepoint, the study benefited from a five-year longitudinal assessment of sleep. While directionality of the relationships between sleep patterns and TSPO levels cannot be determined, it enables meaningful insights into the role of long-term sleep trajectories and their associations with neuroimmune markers. Future research could assess the relative importance of each pathway direction, determining whether targeting sleep or neuroimmune pathways yields greater benefits for brain health.

Taken together, sleep duration emerged as the sleep dimension being most dominantly associated with TSPO levels. If replicated, sleep duration could represent a modifiable marker of neuroimmune risk in aging. Future research could test whether extending sleep duration may be particularly beneficial for individuals with shorter sleep durations and higher TSPO levels in MFC. These investigations could help evaluate the therapeutic potential of interventions to promote brain health and sleep.

## Supplementary Information

Supplementary Material 1

## Data Availability

Data are available upon request from the corresponding authors.
