# Supplementary material for: A [11C]PBR28 PET study on the associations between sleep health and microglial density
Source: J Neuroinflammation. 2025 Nov 14;22:270. doi: 10.1186/s12974-025-03613-1 (PMC12619189; doi:10.1186/s12974-025-03613-1)
Supplement: Supplementary file 1 — Supplementary Material 1 [file 12974_2025_3613_MOESM1_ESM.docx]

**A [^11^C]PBR28 PET study on the associations between sleep health and microglial density**

Leonie JT Balter, Jonatan Malmros, Per Stenkrona, Andrea Varrone, Anton Forsberg, Erik Gustavsson, Cedrique E Mouyobo, Grégoria Kalpouzos, & Goran Papenberg

**SUPPLEMENTARY MATERIALS**

**Table of Contents**

[Supplementary Methods and Results 3](#_Toc212816349)

[Additional imaging details: MRI and quantification of TSPO binding 3](#_Toc212816350)

[Additional details on ROI selection 3](#_Toc212816351)

[Additional genotyping details 4](#_Toc212816352)

[Exploratory analysis: Latent sleep factors 4](#_Toc212816353)

[Additional factor analysis results 5](#_Toc212816354)

[Supplementary Figures 6](#_Toc212816355)

[Figure S1. Distributions of sleep dimension variables 6](#_Toc212816356)

[Figure S2. Distributions of TSPO binding in regions of interest separated by TSPO polymorphism (rs6971) genotype 7](#_Toc212816357)

[Figure S3. Correlation matrix of sleep dimension variables 8](#_Toc212816358)

[Figure S4. Scree plot of the exploratory factor analysis 9](#_Toc212816359)

[Figure S5. Residual plots of the significant correlations between the regions of interest (ROIs) and sleep variables 10](#_Toc212816360)

[Figure S6. Changes in sleep dimensions across timepoints 11](#_Toc212816361)

[Figure S7. Associations between longitudinal changes in sleep and TSPO levels 12](#_Toc212816362)

[Supplemental Tables 14](#_Toc212816363)

[Table S1. TSPO binding by genotype 14](#_Toc212816364)

[Table S2. Associations between TSPO levels, age and sex 15](#_Toc212816365)

[Table S3. Associations between TSPO levels and sleep dimensions 16](#_Toc212816366)

[Table S4. Associations between CRP and sleep dimensions 20](#_Toc212816367)

[Table S5. Associations between CRP and TSPO levels 21](#_Toc212816368)

[Table S6. Associations between sleep factors obtained using exploratory factor analysis and TSPO levels 22](#_Toc212816369)

[Table S7. Associations between longitudinal changes in sleep and TSPO levels 23](#_Toc212816370)

[References 27](#_Toc212816371)

# Supplementary Methods and Results

## Additional imaging details: MRI and quantification of TSPO binding

Structural data were collected using a T1-weighted Multi-Echo Magnetization-Prepared Rapid Acquisition Gradient Echo (MEMPRAGE) ^1^ provided by MGH (Massachusetts General Hospital), **collected** at 1-mm isotropic resolution, with parameters: 176 sagittal slices, TR = 2530 ms, TEs = 1.69, 3.55, 5.41, and 7.27 ms, TI = 1100 ms, flip angle = 7°, FOV = 256 mm, GRAPPA acceleration=2. An individual plaster helmet was created for each participant to minimize head movement during the PET scan. Arterial blood was collected continuously during the first 10 min using an automated blood sampling system (ABSS; Allogg AB, Sweden). In addition, arterial blood samples (2–4 ml) were drawn manually at approximately 2, 4, 6, 8, 10, 15, 20, 25, 30, 40, 50, and 60 min after radioligand injection. Blood sampling is necessary for the quantification of the binding of TSPO binding. The blood sampling protocol is optimized based on accumulated experience from previous analyses of [^11^C]PBR28 data. Radiometabolite analyses were carried out as previously described ^2^. The radiometabolite-corrected plasma input function was created as previously described by correcting the measured radioactivity by the fitted parent fraction ^3^. Image processing and the definition of regions of interest (ROIs) using T1 MR images were performed as described previously ^4^. In short, PET images were processed using an in-house pipeline written and executed in MATLAB (MATLAB r2014b, The MathWorks, Inc.). Individual T1-weighted MR images were segmented using FreeSurfer (FreeSurfer v6.0.0, <http://surfer.nmr.mgh.harvard.edu/>) ^5^. The generated ROIs were applied to the co-registered PET images and time activity curves were extracted. Kinetic modelling was performed using the two-tissue compartment model that has previously been shown to provide reliable estimates of total distribution volume (V_T_) ^2^.

## Additional details on ROI selection

The brain regions were selected based on prior evidence of their involvement in sleep–wake regulation and their relevance to neuroinflammatory processes, including consistent TSPO binding in prior PET literature and links to inflammation-related pathophysiology. The **MFC** was included as it corresponds to the **DLPFC**, a region sensitive to sleep loss ^6^. The **frontal cortex (FC)** ROI is broader, spanning multiple frontal subregions. Including both the FC and MFC allows for examination of both general and more specific frontal associations with TSPO binding. The **caudate** and **putamen** were selected as key components of the **striatum**, a region with known links to inflammation (e.g., ^7^). Moreover, both structures show robust TSPO expression and are implicated in inflammation-related pathophysiology in aging.

## Additional genotyping details

DNA was extracted from peripheral blood samples and stored at Karolinska Institutet Biobank. DNA samples were transferred on PCR plates and sent to the SNP&SEQ Technology Platform, Uppsala University [National Genomics Infrastructure (NGI), SciLifeLab Sweden]. The facility is part of the National Genomics Infrastructure supported by the Swedish Research Council for Infrastructures and Science for Life Laboratory, Sweden. The SNP&SEQ Technology Platform is also supported by the Knut and Alice Wallenberg Foundation. The genotyping was performed using the Illumina Infinium assay ^8,9^ and the results were analyzed using the software GenomeStudio 2.0.3. The TSPO polymorphism (rs6971) was part of the analyzed assay.

## Exploratory analysis: Latent sleep factors

To assess the robustness of the results, we conducted exploratory factor analysis to reduce the dimensionality of sleep characteristics and identify underlying constructs. We included the following variables: sleep duration, sleep quality, sleep insufficiency, non-restorative sleep, daytime fatigue, sleep need, chronotype, nap frequency, and social jetlag. Sleep duration deviation was not included for being intertwined with and derived from sleep duration. Maximum likelihood was used as the estimation method and oblique (oblimin) rotation to allow for correlated factors. Variables with a measure of sampling adequacy (MSA) <0.50 were excluded, as values below this threshold indicate that the variable is not suitable for inclusion. Based on this criterion, social jetlag (MSA = 0.32), nap frequency (MSA = 0.39) and chronotype (MSA = 0.49) were excluded. The overall MSA for the final model was 0.73. Although parallel analysis suggested a single-factor solution, we extracted two factors based on theoretical and empirical evidence that sleep duration and sleep quality are related, but represent distinct dimensions of sleep health ^10–12^, each potentially contributing differently to physiological and psychological outcomes. The extraction of two factors results in the sleep duration and sleep quality factor. We emphasize the exploratory nature of this analysis, particularly given the moderate sample size. Factor scores were extracted using the default regression-based Thurstone method and were subsequently correlated with TSPO levels. The factor scores reflect a weighted combination of all sleep variables. They capture broader latent constructs that include shared variance across multiple sleep-related variables, even those contributing weakly. As such, the sleep duration factor score is not equivalent to the raw sleep duration variable. The factor score instead integrates more complex and diffuse information than sleep duration alone. The *fa* function in the *psych* R Package was used for this analysis.

## ****Additional factor analysis results****

The explained variance in the data was 24% for the poor sleep quality factor and 28% for the short sleep duration factor.

# Supplementary Figures

Figure S1. Distributions of sleep dimension variables. Higher values represent worse levels (e.g., worse sleep quality, worse non-restorative sleep) (A,B C, D, H), greater sleep need and sleep duration in hours (E, J), stronger evening chronotype (F), greater nap frequency (G), greater social jetlag (I), and greater sleep duration deviation in minutes (K) (either longer or shorter than 8 hours).

Figure S2. Distributions of TSPO binding in regions of interest separated by TSPO polymorphism (rs6971) genotype. GG = high affinity binders and AG = medium affinity binders. Low affinity binders (AA) were not recruited; ACC = Anterior Cingulate Cortex. See Table S1 for the means, standard deviations, and ranges.

Figure S3. Correlation matrix of sleep dimension variables, showing Spearman’s correlation coefficients. Colored cells indicate statistically significant correlations (*p* < .05).

Figure S4. Scree plot of the exploratory factor analysis (EFA) using the sleep dimensions.

**
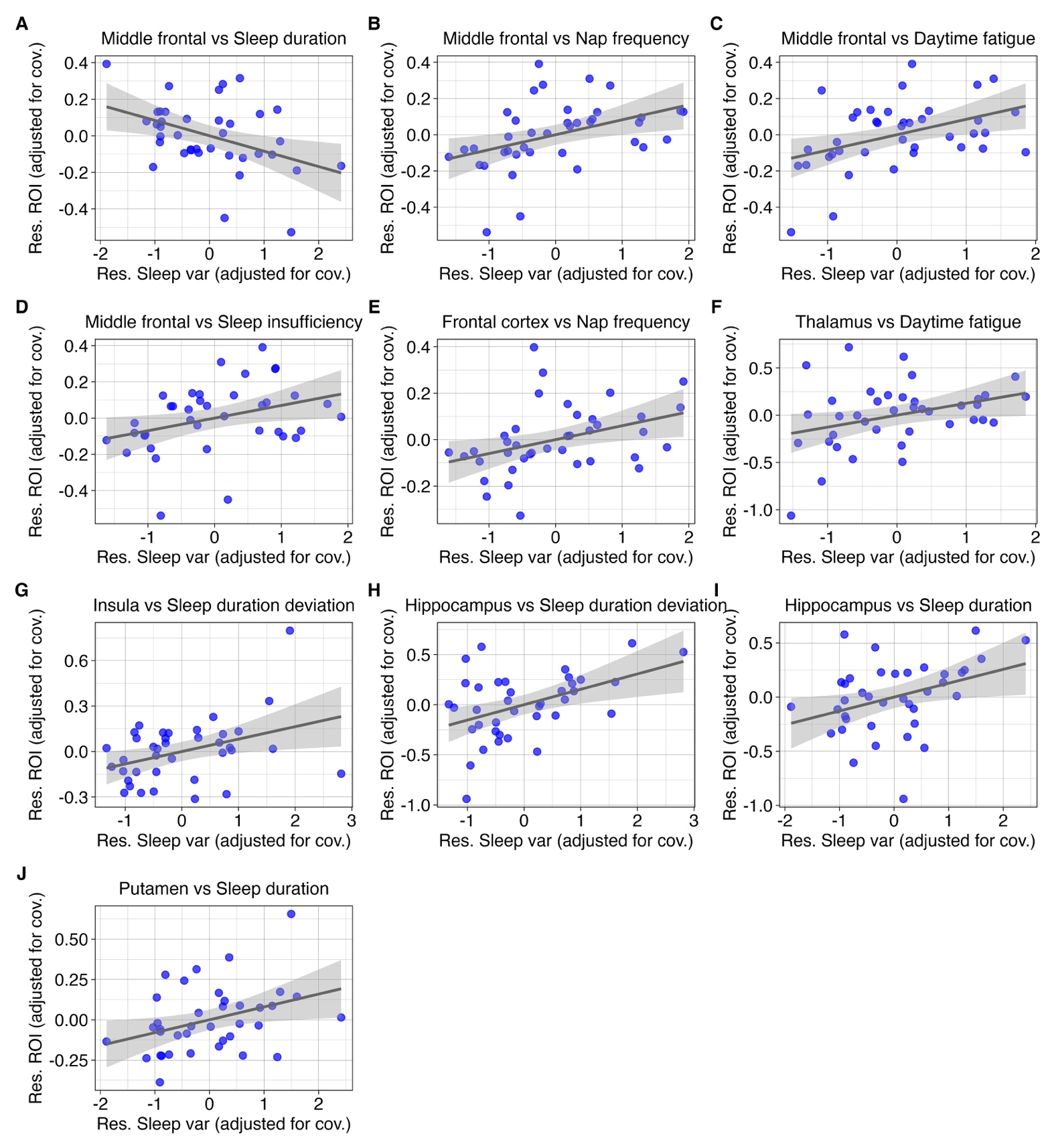
**

Figure S5. Residual plots of the significant correlations between the regions of interest (ROIs) and sleep variables, as shown in Figure 1 (main text). The plots adjusted for age, sex, interval between the PET scan and blood sampling (during which sleep data were collected), and global TSPO. Error bands represent 95% confidence intervals. Abbreviations: Res. = residuals; Cov. = covariates; ROI = Region of interest.


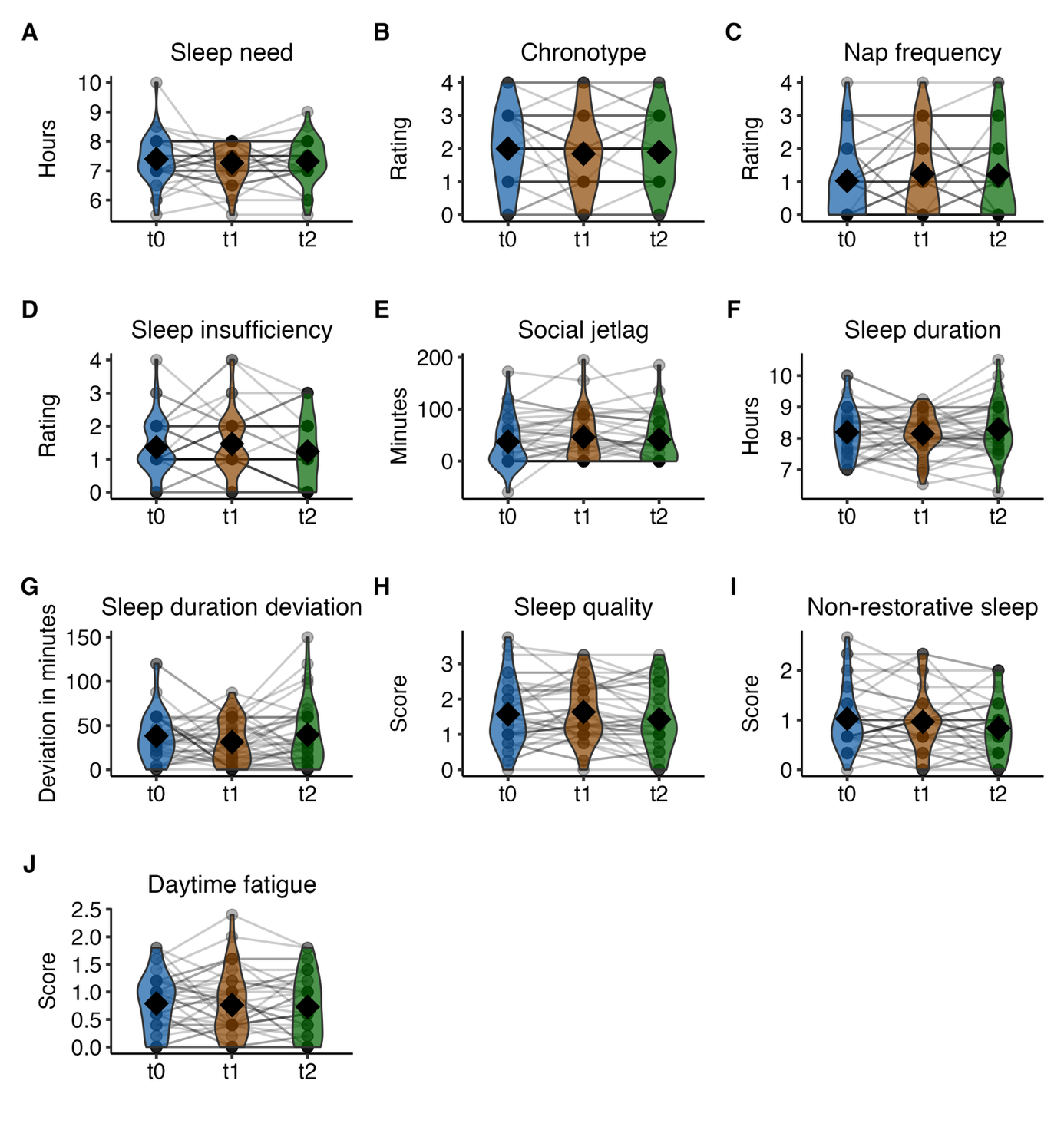


Figure S6. Changes in sleep dimensions across timepoints**.** T0 (Jan 2017 to Dec 2017), T1 (Nov 2019 to Oct 2020), and T2 (Nov 2021 to Oct 2022). Individual data points and trajectories are represented by dots and lines, illustrating changes across the timepoints for each participant. The black diamond markers signify group mean values specific to each timepoint. Higher chronotype values indicate being a stronger evening chronotype; Higher values of sleep duration deviation indicate either a shorter or longer sleep duration than 8 hours. Models adjusted for retirement status at T2 (n = 24 retired, 15 working).

Figure S7. Associations between longitudinal changes in sleep and TSPO levels**,** with standardized coefficients (β), 95% confidence intervals (95% CI), and uncorrected p-values. Models adjusted for sex, age, and retirement status. Models for regional ROIs additionally adjusted for global gray matter volume of distribution (V_T_); the global gray matter model instead adjusted for TSPO rs6971 genotype. Higher values of sleep duration deviation indicate that a larger change in sleep duration deviation over timepoints is associated with higher TSPO levels (measured at T2) in the respective region of interest. MFC = Middle Frontal Cortex; ACC = Anterior Cingulate Cortex.

# Supplemental Tables

Applies to all tables. Uncorrected p-values indicate that no adjustment for multiple comparisons was performed.

Table S1. TSPO binding by genotype**.** Volume of distribution (VT) for high affinity binders (GG) and medium affinity binders (AG), showing mean (M), standard deviation (SD), and range (min, max).

| **ROI** | **GG, *M* (*SD*)**  **range (min, max)** | **AG, *M* (*SD*)**  **range (min, max)** |
| --- | --- | --- |
| Brainstem | 6.05 (1.45)  (3.93, 8.50) | 3.01 (0.64)  (2.07, 4.69) |
| Caudate | 4.11 (1.01)  (2.76, 6.16) | 2.03 (0.51)  (1.20, 3.40) |
| Frontal cortex | 5.43 (1.18)  (3.46, 7.27) | 2.60 (0.52)  (1.75, 4.06) |
| Gray matter | 5.28 (1.16)  (3.40, 7.28) | 2.52 (0.49)  (1.74, 3.83) |
| Hippocampus | 4.93 (1.10)  (2.78, 7.48) | 2.38 (0.52)  (1.50, 3.38) |
| Insula | 5.47 (1.32)  (3.22, 8.52) | 2.57 (0.55)  (1.67, 3.77) |
| MFC | 5.58 (1.22)  (3.51, 7.31) | 2.64 (0.51)  (1.91, 4.12) |
| Putamen | 5.25 (1.22)  (3.46, 8.06) | 2.50 (0.56)  (1.65, 3.64) |
| Thalamus | 6.50 (1.35)  (4.35, 8.96) | 3.12 (0.67)  (2.06, 4.42) |
| ACC | 5.68 (1.42)  (3.27, 9.16) | 2.66 (0.54)  (1.65, 4.09) |

*Note.* MFC = Middle Frontal Cortex; ACC = Anterior Cingulate Cortex

Table S2. Associations between TSPO levels, age and sex. Bold values indicate statistically significant results (p < .05).

|  | **Age**  *M* = 66.7  *SD* = 8.9  Range = 50-81 | | | **Sex**  20 male (coded as 0)  19 female (coded as 1) | | |
| --- | --- | --- | --- | --- | --- | --- |
|  | **r_s_ partial** | **statistic** | **Uncorrected p-value** | **b** | **95% CI** | **Uncorrected p-value** |
| Brainstem | -0.002 | -0.01 | 0.989 | 0.13 | -0.10, 0.25 | 0.255 |
| Caudate | **-0.481** | **-3.29** | **0.002** | 0.23 | -0.00, 0.46 | 0.054 |
| Frontal cortex | 0.090 | 0.54 | 0.591 | -0.07 | -0.17, 0.03 | 0.164 |
| Hippocampus | -0.142 | -0.86 | 0.394 | 0.02 | -0.23, 0.28 | 0.844 |
| Insula | **-0.337** | **-2.15** | **0.038** | -0.00 | -0.17, 0.16 | 0.963 |
| MFC | 0.059 | 0.35 | 0.725 | -0.06 | -0.19, 0.07 | 0.385 |
| Putamen | 0.121 | 0.73 | 0.469 | **-0.23** | **-0.37, -0.08** | **0.003** |
| Thalamus | -0.052 | -0.31 | 0.756 | -0.21 | -0.45, 0.03 | 0.088 |
| ACC | 0.043 | 0.25 | 0.800 | -0.06 | -0.29, 0.17 | 0.618 |
| Gray matter | -0.200 | -1.22 | 0.229 | 0.20 | -0.39, 0.78 | 0.498 |

Note. Age was analyzed using partial correlations. Sex was analyzed using linear regression. All analyses adjusted for global TSPO binding in gray matter. The model assessing global gray matter adjusted for TSPO genotype.

Table S3. Associations between TSPO levels and sleep dimensions**,** with standardized coefficients (β), raw and bootstrapped 95% confidence intervals (CI) are shown. Bold values indicate statistically significant results (p < .05, or 95% CI not containing 0).

| **Sleep duration** | | | | |
| --- | --- | --- | --- | --- |
| **ROI** | **β** | **Raw 95% CI** | **Uncorrected p-value** | **Bootstrapped 95% CI** |
| Caudate | -0.50 | -1.73, 0.73 | 0.417 | -1.84, 1.19 |
| Frontal cortex | -1.38 | -3.64, 0.88 | 0.223 | -4.13, 0.83 |
| Hippocampus | **1.03** | **0.08, 1.98** | **0.035** | **0.01, 2.46** |
| Insula | 0.36 | -1.27, 1.99 | 0.656 | -2.84, 1.67 |
| MFC | **-2.09** | **-3.72, -0.46** | **0.013** | **-3.82, -0.35** |
| Putamen | **1.66** | **0.13, 3.19** | **0.035** | **0.25, 3.16** |
| Thalamus | 0.08 | -0.94, 1.11 | 0.869 | -1.04, 1.91 |
| ACC | 0.67 | -0.39, 1.73 | 0.208 | -0.73, 1.72 |
| Brainstem | -0.36 | -1.45, 0.73 | 0.504 | -1.37, 0.79 |
| Gray matter | 0.10 | -0.31, 0.51 | 0.625 | -0.41, 0.51 |
|  |  |  |  |  |
| **Nap frequency** | | | | |
| Caudate | -0.16 | -1.18, 0.87 | 0.756 | -1.84, 0.65 |
| Frontal cortex | **2.42** | **0.34, 4.50** | **0.024** | **0.54, 5.43** |
| Hippocampus | -0.54 | -1.41, 0.32 | 0.210 | -1.33, 0.49 |
| Insula | -0.67 | -2.04, 0.69 | 0.322 | -1.50, 1.26 |
| MFC | **2.04** | **0.44, 3.65** | **0.014** | **0.93, 3.87** |
| Putamen | -0.62 | -2.20, 0.97 | 0.434 | -1.77, 1.11 |
| Thalamus | 0.27 | -0.67, 1.22 | 0.560 | -0.90, 1.22 |
| ACC | 0.01 | -0.98, 1.00 | 0.987 | -0.86, 1.67 |
| Brainstem | -0.52 | -1.58, 0.54 | 0.324 | -1.63, 0.52 |
| Gray matter | -0.20 | -0.58, 0.19 | 0.301 | -0.64, 0.22 |
|  |  |  |  |  |
| **Daytime fatigue** | | | | |
| Caudate | 0.37 | -0.64, 1.39 | 0.457 | -0.82, 1.28 |
| Frontal cortex | 1.41 | -0.78, 3.60 | 0.199 | -0.79, 4.26 |
| Hippocampus | -0.15 | -1.03, 0.73 | 0.734 | -1.22, 0.74 |
| Insula | -0.53 | -1.89, 0.84 | 0.438 | -1.74, 1.70 |
| MFC | **2.10** | **0.50, 3.69** | **0.011** | **0.65, 3.53** |
| Putamen | -0.13 | -1.72, 1.46 | 0.870 | -1.72, 2.63 |
| Thalamus | **0.91** | **0.02, 1.80** | **0.046** | -0.07, 1.93 |
| ACC | 0.17 | -0.81, 1.16 | 0.723 | -0.79, 1.92 |
| Brainstem | -0.28 | -1.35, 0.78 | 0.594 | -1.44, 0.91 |
| Gray matter | 0.14 | -0.24, 0.53 | 0.458 | -0.27, 0.71 |

**Table S3.** Continued

| **Social jetlag** | | | | |
| --- | --- | --- | --- | --- |
| **ROI** | **β** | **Raw 95% CI** | **Uncorrected p-value** | **Bootstrapped 95% CI** |
| Caudate | 0.41 | -0.77, 1.59 | 0.482 | -0.58, 1.78 |
| Frontal cortex | -0.96 | -3.04, 1.12 | 0.355 | -3.83, 0.66 |
| Hippocampus | 0.27 | -0.69, 1.22 | 0.574 | -0.53, 1.44 |
| Insula | 0.65 | -0.90, 2.20 | 0.398 | -0.45, 4.13 |
| MFC | 0.00 | -1.65, 1.64 | 0.997 | -2.32, 1.29 |
| Putamen | 0.00 | -1.53, 1.52 | 0.997 | -1.80, 2.20 |
| Thalamus | -0.37 | -1.30, 0.56 | 0.422 | -1.50, 0.34 |
| ACC | -0.01 | -0.99, 0.97 | 0.982 | -1.22, 0.90 |
| Brainstem | -0.84 | -1.83, 0.15 | 0.093 | **-1.84, -0.08** |
| Gray matter | 0.17 | -0.23, 0.57 | 0.386 | -0.22, 0.54 |
|  |  |  |  |  |
| **Sleep need** | | | | |
| Caudate | -0.85 | -1.84, 0.14 | 0.090 | -1.97, 0.19 |
| Frontal cortex | 0.11 | -2.16, 2.38 | 0.922 | -2.62, 2.96 |
| Hippocampus | 0.63 | -0.23, 1.49 | 0.148 | -0.14, 1.46 |
| Insula | 0.55 | -0.83, 1.93 | 0.424 | -2.47, 1.50 |
| MFC | -0.41 | -2.18, 1.36 | 0.641 | -2.60, 2.26 |
| Putamen | -0.24 | -1.85, 1.37 | 0.765 | -3.24, 1.38 |
| Thalamus | -0.43 | -1.38, 0.52 | 0.363 | -1.29, 0.79 |
| ACC | 0.78 | -0.19, 1.74 | 0.112 | -0.56, 1.84 |
| Brainstem | -0.13 | -1.21, 0.95 | 0.808 | -1.38, 0.84 |
| Gray matter | 0.22 | -0.17, 0.60 | 0.266 | -0.25, 0.60 |
|  |  |  |  |  |
| **Sleep duration deviation** | | | | |
| Caudate | -0.69 | -1.95, 0.56 | 0.269 | -2.09, 0.90 |
| Frontal cortex | -1.61 | -3.92, 0.70 | 0.166 | -3.89, 0.92 |
| Hippocampus | **1.30** | **0.37, 2.24** | **0.008** | **0.14, 2.46** |
| Insula | **1.80** | **0.24, 3.35** | **0.025** | -0.56, 3.05 |
| MFC | -1.29 | -3.08, 0.50 | 0.151 | -3.44, 1.14 |
| Putamen | 1.36 | -0.26, 2.97 | 0.098 | -0.87, 2.72 |
| Thalamus | 0.55 | -0.49, 1.58 | 0.289 | -0.94, 2.44 |
| ACC | 1.03 | -0.02, 2.07 | 0.053 | -0.22, 2.25 |
| Brainstem | 0.47 | -0.64, 1.58 | 0.394 | -0.70, 1.44 |
| Gray matter | -0.01 | -0.44, 0.41 | 0.951 | -0.74, 0.52 |

**Table S3.** Continued

| **Sleep quality** | | | | |
| --- | --- | --- | --- | --- |
| **ROI** | **β** | **Raw 95% CI** | **Uncorrected p-value** | **Bootstrapped 95% CI** |
| Caudate | 0.51 | -0.56, 1.59 | 0.338 | -0.30, 1.61 |
| Frontal cortex | 1.50 | -0.84, 3.83 | 0.202 | -0.69, 4.83 |
| Hippocampus | -0.36 | -1.29, 0.57 | 0.437 | -1.42, 0.50 |
| Insula | 0.15 | -1.32, 1.63 | 0.833 | -1.16, 2.28 |
| MFC | 1.25 | -0.57, 3.08 | 0.171 | -0.35, 3.35 |
| Putamen | 1.07 | -0.59, 2.73 | 0.199 | -0.55, 3.53 |
| Thalamus | 0.77 | -0.21, 1.75 | 0.120 | -0.11, 1.78 |
| ACC | 0.34 | -0.67, 1.36 | 0.496 | -0.42, 1.91 |
| Brainstem | -0.27 | -1.41, 0.87 | 0.634 | -1.53, 0.76 |
| Gray matter | -0.02 | -0.43, 0.39 | 0.934 | -0.39, 0.44 |
|  |  |  |  |  |
| **Chronotype** | | | | |
| Caudate | -0.55 | -1.57, 0.48 | 0.288 | -1.62, 0.21 |
| Frontal cortex | -0.16 | -2.45, 2.14 | 0.889 | -3.34, 1.64 |
| Hippocampus | -0.14 | -1.04, 0.77 | 0.761 | -0.95, 0.83 |
| Insula | -0.01 | -1.42, 1.40 | 0.986 | -1.03, 1.50 |
| MFC | -0.08 | -1.88, 1.72 | 0.929 | -2.33, 1.33 |
| Putamen | 0.10 | -1.53, 1.73 | 0.899 | -1.00, 2.02 |
| Thalamus | 0.41 | -0.55, 1.38 | 0.388 | -0.59, 1.70 |
| ACC | -0.74 | -1.71, 0.23 | 0.130 | -1.96, 0.07 |
| Brainstem | 0.35 | -0.73, 1.44 | 0.512 | -0.57, 1.60 |
| Gray matter | 0.21 | -0.18, 0.61 | 0.279 | -0.15, 0.64 |
|  |  |  |  |  |
| **Non-restorative sleep** | | | | |
| Caudate | 0.81 | -0.20, 1.82 | 0.111 | **0.07, 1.84** |
| Frontal cortex | 0.60 | -1.69, 2.90 | 0.595 | -1.26, 3.58 |
| Hippocampus | -0.17 | -1.07, 0.74 | 0.712 | -1.37, 0.70 |
| Insula | 0.10 | -1.31, 1.52 | 0.882 | -1.20, 1.94 |
| MFC | 0.88 | -0.89, 2.66 | 0.318 | -0.49, 2.92 |
| Putamen | 0.48 | -1.14, 2.11 | 0.549 | -1.18, 2.00 |
| Thalamus | 0.76 | -0.18, 1.70 | 0.108 | **0.10, 1.74** |
| ACC | 0.04 | -0.95, 1.03 | 0.931 | -0.92, 1.00 |
| Brainstem | 0.83 | -0.23, 1.89 | 0.120 | -0.11, 1.80 |
| Gray matter | 0.15 | -0.23, 0.54 | 0.430 | -0.14, 0.48 |

**Table S3.** Continued

| **Sleep insufficiency** | | | | |  |
| --- | --- | --- | --- | --- | --- |
| **ROI** | **β** | **Raw 95% CI** | **Uncorrected p-value** | **Bootstrapped 95% CI** | |
| Caudate | 0.36 | -0.65, 1.37 | 0.476 | -0.34, 1.28 | |
| Frontal cortex | 1.82 | -0.33, 3.96 | 0.094 | **0.31, 3.46** | |
| Hippocampus | -0.53 | -1.39, 0.33 | 0.218 | -1.32, 0.15 | |
| Insula | 0.10 | -1.28, 1.48 | 0.882 | -0.70, 2.33 | |
| MFC | **1.71** | **0.06, 3.35** | **0.042** | **0.54, 3.19** | |
| Putamen | -0.22 | -1.81, 1.37 | 0.781 | -1.95, 1.67 | |
| Thalamus | 0.17 | -0.77, 1.12 | 0.712 | -0.75, 1.02 | |
| ACC | -0.19 | -1.17, 0.78 | 0.687 | -1.09, 0.92 | |
| Brainstem | -0.41 | -1.47, 0.65 | 0.438 | -1.13, 0.36 | |
| Gray matter | -0.12 | -0.50, 0.27 | 0.539 | -0.45, 0.29 | |

*Note.* MFC = Middle Frontal Cortex; ACC = Anterior Cingulate Cortex

Table S4. Associations between CRP and sleep dimensions with standardized coefficients (β), raw and bootstrapped 95% confidence intervals (CI), and uncorrected p-values. Bold values indicate statistically significant results (p < .05, or 95% CI not containing 0).

| **CRP and sleep dimension variables** | | | | |
| --- | --- | --- | --- | --- |
| **Sleep dimension T2** | **β** | **Raw 95% CI** | **Uncorrected p-value** | **Bootstrapped 95% CI** |
| Nap frequency | 0.29 | -0.02, 0.59 | 0.064 | **0.06, 0.61** |
| Social jetlag | -0.07 | -0.42, 0.27 | 0.673 | -0.47, 0.26 |
| Non-restorative sleep | 0.20 | -0.11, 0.51 | 0.200 | -0.11, 0.48 |
| Daytime fatigue | 0.19 | -0.13, 0.51 | 0.233 | -0.12, 0.55 |
| Sleep duration deviation | -0.12 | -0.41, 0.16 | 0.395 | -0.44, 0.15 |
| Sleep insufficiency | 0.14 | -0.17, 0.46 | 0.362 | -0.12, 0.44 |
| Sleep quality | 0.04 | -0.27, 0.35 | 0.787 | -0.25, 0.31 |
| Chronotype | -0.02 | -0.33, 0.3 | 0.921 | -0.30, 0.31 |
| Sleep need | -0.11 | -0.42, 0.19 | 0.446 | -0.48, 0.12 |
| Sleep duration | -0.23 | -0.52, 0.06 | 0.121 | **-0.48, -0.02** |

Table S5. Associations between CRP and TSPO levels, with unstandardized coefficients (b), raw and bootstrapped 95% confidence intervals, and uncorrected p-values, corrected for sex, age, interval between the PET scan and blood sampling (during which sleep data were collected), and TSPO genotype (for GM) and gray matter volume of distribution (VT) (for all other ROIs).

| **Peripheral CRP and TSPO level in each ROI** | | | | |
| --- | --- | --- | --- | --- |
| **ROI** | **b** | **Raw 95% CI** | **Uncorrected p-value** | **Bootstrapped 95% CI** |
| Caudate | -0.10 | -1.06, 0.85 | 0.826 | -1.97, 1.24 |
| Frontal cortex | 0.35 | -1.71, 2.41 | 0.733 | -1.91, 2.85 |
| Hippocampus | -0.57 | -1.36, 0.22 | 0.150 | -1.44, 0.29 |
| Insula | -0.31 | -1.60, 0.98 | 0.626 | -2.20, 2.21 |
| MFC | 1.19 | -0.37, 2.75 | 0.131 | -0.09, 2.93 |
| Putamen | -0.33 | -1.81, 1.16 | 0.657 | -2.20, 0.88 |
| Thalamus | 0.42 | -0.46, 1.29 | 0.340 | -0.57, 1.51 |
| ACC | -0.30 | -1.22, 0.62 | 0.508 | -1.80, 0.69 |
| Brainstem | 0.39 | -0.63, 1.41 | 0.441 | -0.60, 1.27 |
| Gray matter | 0.05 | -0.30, 0.41 | 0.765 | -0.33, 0.48 |

*Note.* MFC = Middle Frontal Cortex; ACC = Anterior Cingulate Cortex

Table S6. Associations between sleep factors obtained using exploratory factor analysis and TSPO levels**,** with unstandardized coefficients (b), raw and bootstrapped 95% confidence intervals (CI), and uncorrected p-values, adjusted for sex, age, interval between the PET scan and blood sampling (during which sleep data were collected), and TSPO genotype (for GM) and gray matter volume of distribution (V_T_) (for all other ROIs). Bold values indicate statistically significant results (*p* < .05, or 95% CI not containing 0).

|  | **Sleep factors (EFA) and TSPO level in each ROI** | | | | |
| --- | --- | --- | --- | --- | --- |
| **ROI** | **Factor** | **b** | **Raw 95% CI** | **Uncorrected p-value** | **Bootstrapped 95% CI** |
| Caudate | Poor sleep quality (Factor 1) | 0.85 | -0.43, 2.13 | 0.186 | -0.32, 1.84 |
|  | Short sleep duration (Factor 2) | 0.61 | -0.53, 1.76 | 0.285 | -0.43, 1.60 |
| Frontal cortex | Poor sleep quality (Factor 1) | 1.45 | -0.95, 3.85 | 0.227 | -0.82, 4.74 |
|  | Short sleep duration (Factor 2) | 1.74 | -0.34, 3.82 | 0.097 | 0.37, 3.57 |
| Hippocampus | Poor sleep quality (Factor 1) | -0.53 | -1.6, 0.53 | 0.314 | -1.88, 0.40 |
|  | Short sleep duration (Factor 2) | -0.71 | -1.63, 0.21 | 0.128 | -1.64, 0.08 |
| Insula | Poor sleep quality (Factor 1) | 0.06 | -1.68, 1.79 | 0.946 | -1.47, 2.69 |
|  | Short sleep duration (Factor 2) | 0.02 | -1.51, 1.56 | 0.976 | -1.13, 2.51 |
| MFC | Poor sleep quality (Factor 1) | 1.25 | -0.60, 3.10 | 0.178 | -0.35, 3.39 |
|  | Short sleep duration (Factor 2) | **1.76** | **0.20, 3.32** | **0.028** | **0.62, 3.15** |
| Putamen | Poor sleep quality (Factor 1) | 1.07 | -0.63, 2.77 | 0.208 | -0.57, 3.39 |
|  | Short sleep duration (Factor 2) | -0.33 | -1.87, 1.21 | 0.665 | -2.02, 1.57 |
| Thalamus | Poor sleep quality (Factor 1) | 0.91 | -0.12, 1.94 | 0.083 | 0.01, 1.84 |
|  | Short sleep duration (Factor 2) | 0.26 | -0.7, 1.21 | 0.588 | -0.75, 1.19 |
| ACC | Poor sleep quality (Factor 1) | 0.35 | -0.75, 1.46 | 0.519 | -0.45, 2.25 |
|  | Short sleep duration (Factor 2) | -0.27 | -1.27, 0.73 | 0.586 | -1.18, 0.84 |
| Brainstem | Poor sleep quality (Factor 1) | -0.26 | -1.42, 0.89 | 0.644 | -1.55, 0.91 |
|  | Short sleep duration (Factor 2) | -0.25 | -1.27, 0.77 | 0.624 | -1.08, 0.51 |
| Gray matter | Poor sleep quality (Factor 1) | 0.01 | -0.43, 0.45 | 0.964 | -0.34, 0.51 |
|  | Short sleep duration (Factor 2) | -0.09 | -0.48, 0.29 | 0.626 | -0.44, 0.37 |

*Note.* MFC = Middle Frontal Cortex; ACC = Anterior Cingulate Cortex

Table S7. Associations between longitudinal changes in sleep and TSPO levels, with unstandardized coefficients (b), 95% confidence intervals (95% CI), and uncorrected p-values, adjusted for sex, age, retirement status, and TSPO genotype (for GM) or gray matter volume of distribution (VT) (for all other ROIs). Bold values indicate statistically significant results (p < .05).

| **Sleep duration** | | | | |
| --- | --- | --- | --- | --- |
| **ROI** | **b** | **95% CI** | | **Uncorrected p-value** |
|  |  | **Lower** | **Upper** |  |
| Timepoint x Caudate | -0.02 | -0.14 | 0.11 | 0.776 |
| Timepoint x Frontal Cortex | 0.00 | -0.09 | 0.10 | 0.923 |
| Timepoint x Hippocampus | 0.02 | -0.08 | 0.11 | 0.764 |
| Timepoint x Insula | 0.01 | -0.08 | 0.09 | 0.896 |
| Timepoint x MFC | 0.00 | -0.09 | 0.09 | 0.970 |
| Timepoint x Putamen | 0.02 | -0.07 | 0.11 | 0.701 |
| Timepoint x Thalamus | 0.01 | -0.07 | 0.09 | 0.844 |
| Timepoint x ACC | 0.01 | -0.08 | 0.09 | 0.840 |
| Timepoint x Brainstem | 0.00 | -0.08 | 0.08 | 0.998 |
| Timepoint x Gray matter | 0.01 | -0.09 | 0.10 | 0.859 |
|  |  |  | |  |
| **Nap frequency** | | | | |
| Timepoint x Caudate | 0.02 | -0.08 | 0.11 | 0.742 |
| Timepoint x Frontal Cortex | 0.01 | -0.06 | 0.08 | 0.789 |
| Timepoint x Hippocampus | 0.00 | -0.08 | 0.08 | 0.986 |
| Timepoint x Insula | 0.00 | -0.07 | 0.07 | 0.954 |
| Timepoint x MFC | 0.01 | -0.06 | 0.08 | 0.714 |
| Timepoint x Putamen | 0.01 | -0.06 | 0.08 | 0.785 |
| Timepoint x Thalamus | 0.01 | -0.05 | 0.07 | 0.722 |
| Timepoint x ACC | 0.01 | -0.06 | 0.07 | 0.841 |
| Timepoint x Brainstem | 0.00 | -0.06 | 0.07 | 0.972 |
| Timepoint x Gray matter | 0.01 | -0.06 | 0.08 | 0.819 |
|  |  |  | |  |
| **Daytime fatigue** | | | | |
| Timepoint x Caudate | 0.03 | -0.08 | 0.13 | 0.631 |
| Timepoint x Frontal Cortex | 0.01 | -0.07 | 0.09 | 0.807 |
| Timepoint x Hippocampus | 0.01 | -0.08 | 0.09 | 0.903 |
| Timepoint x Insula | 0.01 | -0.07 | 0.08 | 0.881 |
| Timepoint x MFC | 0.01 | -0.06 | 0.09 | 0.709 |
| Timepoint x Putamen | 0.01 | -0.08 | 0.09 | 0.897 |
| Timepoint x Thalamus | 0.01 | -0.06 | 0.08 | 0.765 |
| Timepoint x ACC | 0.01 | -0.06 | 0.09 | 0.766 |
| Timepoint x Brainstem | 0.00 | -0.07 | 0.07 | 0.983 |
| Timepoint x Gray matter | 0.01 | -0.07 | 0.09 | 0.823 |

**Table S7.** Continued

| **Social jetlag** | | | | |
| --- | --- | --- | --- | --- |
| **ROI** | **b** | **95% CI** | | **Uncorrected p-value** |
|  |  | **Lower** | **Upper** |  |
| Timepoint x Caudate | -0.02 | -0.13 | 0.08 | 0.639 |
| Timepoint x Frontal Cortex | -0.03 | -0.11 | 0.05 | 0.418 |
| Timepoint x Hippocampus | -0.03 | -0.11 | 0.06 | 0.529 |
| Timepoint x Insula | -0.03 | -0.10 | 0.04 | 0.431 |
| Timepoint x MFC | -0.03 | -0.10 | 0.05 | 0.467 |
| Timepoint x Putamen | -0.04 | -0.12 | 0.04 | 0.332 |
| Timepoint x Thalamus | -0.03 | -0.09 | 0.04 | 0.455 |
| Timepoint x ACC | -0.03 | -0.10 | 0.04 | 0.373 |
| Timepoint x Brainstem | -0.03 | -0.09 | 0.04 | 0.472 |
| Timepoint x Gray matter | -0.03 | -0.11 | 0.05 | 0.434 |
|  |  |  | |  |
| **Sleep need** | | | | |
| Timepoint x Caudate | 0.05 | -0.08 | 0.19 | 0.460 |
| Timepoint x Frontal Cortex | 0.06 | -0.05 | 0.16 | 0.276 |
| Timepoint x Hippocampus | 0.08 | -0.04 | 0.19 | 0.189 |
| Timepoint x Insula | 0.05 | -0.05 | 0.15 | 0.314 |
| Timepoint x MFC | 0.06 | -0.04 | 0.16 | 0.266 |
| Timepoint x Putamen | 0.05 | -0.05 | 0.16 | 0.348 |
| Timepoint x Thalamus | 0.05 | -0.04 | 0.14 | 0.254 |
| Timepoint x ACC | 0.06 | -0.04 | 0.15 | 0.259 |
| Timepoint x Brainstem | 0.06 | -0.04 | 0.15 | 0.243 |
| Timepoint x Gray matter | 0.06 | -0.05 | 0.17 | 0.269 |
|  |  |  | |  |
| **Sleep duration deviation** | | | | |
| Timepoint x Caudate | **0.16** | **0.01** | **0.31** | **0.046** |
| Timepoint x Frontal Cortex | 0.12 | 0.00 | 0.23 | 0.052 |
| Timepoint x Hippocampus | **0.14** | **0.02** | **0.26** | **0.027** |
| Timepoint x Insula | **0.12** | **0.01** | **0.23** | **0.035** |
| Timepoint x MFC | **0.11** | **0.00** | **0.22** | **0.048** |
| Timepoint x Putamen | **0.13** | **0.02** | **0.25** | **0.029** |
| Timepoint x Thalamus | **0.11** | **0.02** | **0.21** | **0.025** |
| Timepoint x ACC | **0.12** | **0.01** | **0.22** | **0.029** |
| Timepoint x Brainstem | **0.11** | **0.01** | **0.21** | **0.043** |
| Timepoint x Gray matter | **0.13** | **0.01** | **0.25** | **0.037** |

**Table S7.** Continued

| **Sleep quality** | | | | |
| --- | --- | --- | --- | --- |
| **ROI** | **b** | **95% CI** | | **Uncorrected p-value** |
|  |  | **Lower** | **Upper** |  |
| Timepoint x Caudate | 0.04 | -0.04 | 0.12 | 0.360 |
| Timepoint x Frontal Cortex | 0.04 | -0.02 | 0.11 | 0.207 |
| Timepoint x Hippocampus | 0.03 | -0.04 | 0.10 | 0.383 |
| Timepoint x Insula | 0.04 | -0.03 | 0.10 | 0.273 |
| Timepoint x MFC | 0.04 | -0.02 | 0.10 | 0.213 |
| Timepoint x Putamen | 0.04 | -0.02 | 0.11 | 0.188 |
| Timepoint x Thalamus | 0.04 | -0.02 | 0.09 | 0.206 |
| Timepoint x ACC | 0.03 | -0.03 | 0.09 | 0.299 |
| Timepoint x Brainstem | 0.03 | -0.03 | 0.09 | 0.270 |
| Timepoint x Gray matter | 0.04 | -0.03 | 0.11 | 0.246 |
|  |  |  | |  |
| **Chronotype** | | | | |
| Timepoint x Caudate | -0.02 | -0.10 | 0.06 | 0.621 |
| Timepoint x Frontal Cortex | -0.01 | -0.07 | 0.05 | 0.700 |
| Timepoint x Hippocampus | -0.03 | -0.09 | 0.04 | 0.403 |
| Timepoint x Insula | -0.01 | -0.07 | 0.04 | 0.633 |
| Timepoint x MFC | -0.01 | -0.07 | 0.05 | 0.667 |
| Timepoint x Putamen | -0.01 | -0.07 | 0.05 | 0.731 |
| Timepoint x Thalamus | -0.01 | -0.06 | 0.04 | 0.670 |
| Timepoint x ACC | -0.03 | -0.08 | 0.02 | 0.253 |
| Timepoint x Brainstem | -0.01 | -0.06 | 0.04 | 0.703 |
| Timepoint x Gray matter | -0.02 | -0.08 | 0.05 | 0.635 |
|  |  |  | |  |
| **Non-restorative sleep** | | | | |
| Timepoint x Caudate | 0.01 | -0.08 | 0.11 | 0.790 |
| Timepoint x Frontal Cortex | 0.01 | -0.06 | 0.09 | 0.752 |
| Timepoint x Hippocampus | 0.00 | -0.08 | 0.09 | 0.928 |
| Timepoint x Insula | 0.01 | -0.06 | 0.08 | 0.781 |
| Timepoint x MFC | 0.01 | -0.06 | 0.08 | 0.792 |
| Timepoint x Putamen | 0.02 | -0.06 | 0.09 | 0.702 |
| Timepoint x Thalamus | 0.01 | -0.06 | 0.07 | 0.826 |
| Timepoint x ACC | 0.01 | -0.06 | 0.08 | 0.717 |
| Timepoint x Brainstem | 0.02 | -0.05 | 0.09 | 0.575 |
| Timepoint x Gray matter | 0.01 | -0.07 | 0.09 | 0.823 |

**Table S7.** Continued

| **Sleep insufficiency** | | | | |
| --- | --- | --- | --- | --- |
| **ROI** | **b** | **95% CI** | | **Uncorrected p-value** |
|  |  | **Lower** | **Upper** |  |
| Timepoint x Caudate | 0.04 | -0.06 | 0.14 | 0.453 |
| Timepoint x Frontal Cortex | 0.04 | -0.03 | 0.12 | 0.288 |
| Timepoint x Hippocampus | 0.02 | -0.06 | 0.11 | 0.641 |
| Timepoint x Insula | 0.04 | -0.04 | 0.11 | 0.341 |
| Timepoint x MFC | 0.04 | -0.03 | 0.11 | 0.295 |
| Timepoint x Putamen | 0.04 | -0.04 | 0.12 | 0.340 |
| Timepoint x Thalamus | 0.03 | -0.04 | 0.10 | 0.371 |
| Timepoint x ACC | 0.03 | -0.04 | 0.10 | 0.347 |
| Timepoint x Brainstem | 0.03 | -0.04 | 0.10 | 0.362 |
| Timepoint x Gray matter | 0.04 | -0.06 | 0.14 | 0.453 |

*Note.* MFC = Middle Frontal Cortex; ACC = Anterior Cingulate Cortex

# References

1. Van Der Kouwe AJW, Benner T, Salat DH, Fischl B. Brain morphometry with multiecho MPRAGE. NeuroImage. 2008 Apr;40(2):559–69.

2. Collste K, Forsberg A, Varrone A, Amini N, Aeinehband S, Yakushev I, et al. Test–retest reproducibility of [11C]PBR28 binding to TSPO in healthy control subjects. Eur J Nucl Med Mol Imaging. 2016 Jan;43(1):173–83.

3. Forsberg A, Lampa J, Estelius J, Cervenka S, Farde L, Halldin C, et al. Disease activity in rheumatoid arthritis is inversely related to cerebral TSPO binding assessed by [11C]PBR28 positron emission tomography. Journal of Neuroimmunology. 2019 Sept;334:577000.

4. Brumberg J, Aarnio R, Forsberg A, Marjamäki P, Kerstens V, Moein MM, et al. Quantification of the purinergic P2X _7_ receptor with [ ^11^ C]SMW139 improves through correction for brain-penetrating radiometabolites. J Cereb Blood Flow Metab. 2023 Feb;43(2):258–68.

5. Fischl B, Salat DH, Busa E, Albert M, Dieterich M, Haselgrove C, et al. Whole Brain Segmentation. Neuron. 2002 Jan;33(3):341–55.

6. Krause AJ, Simon EB, Mander BA, Greer SM, Saletin JM, Goldstein-Piekarski AN, et al. The sleep-deprived human brain. Nature Reviews Neuroscience. 2017.

7. Felger JC, Li Z, Haroon E, Woolwine BJ, Jung MY, Hu X, et al. Inflammation is associated with decreased functional connectivity within corticostriatal reward circuitry in depression. Molecular Psychiatry. 2016;21(10):1358–65.

8. Gunderson KL, Steemers FJ, Lee G, Mendoza LG, Chee MS. A genome-wide scalable SNP genotyping assay using microarray technology. Nat Genet. 2005 May;37(5):549–54.

9. Steemers FJ, Chang W, Lee G, Barker DL, Shen R, Gunderson KL. Whole-genome genotyping with the single-base extension assay. Nat Methods. 2006 Jan;3(1):31–3.

10. Buysse DJ. Sleep health: can we define It? does it matter? Sleep. 2014;37(1):9–17.

11. Pilcher JJ, Ginter DR, Sadowsky B. Sleep quality versus sleep quantity: Relationships between sleep and measures of health, well-being and sleepiness in college students. Journal of Psychosomatic Research. 1997;42(6):583–96.

12. Van Elk F, Loef B, Proper KI, Burdorf A, Robroek SJW, Oude Hengel KM. Sleep quality, sleep duration, and sleep disturbances among hospital night workers: a prospective cohort study. Int Arch Occup Environ Health. 2024 Mar;97(2):179–88.
